# Do publicly funded community physical activity programs for middle-aged and older adults in Ireland work?

**DOI:** 10.1007/s10433-025-00847-z

**Published:** 2025-03-18

**Authors:** Enrique García Bengoechea, Catherine B. Woods

**Affiliations:** Department of Physical Education and Sport Sciences, Physical Activity for Health Research Centre, Health Research Institute, University of Limerick, Limerick, Ireland

**Keywords:** Healthy ageing, Physical activity, Program evaluation, Intervention, Community-based

## Abstract

To strengthen practice-based evidence, pragmatic, yet rigorous, evaluation of real-world programs is necessary. This study sought to add to the evidence for the effectiveness of physical activity programs for middle-aged and older adults offered by publicly funded local sports partnerships (LSPs) in Ireland. We analysed data from 468 individuals aged 50 + years, who took part in the Move for Life cluster randomised feasibility trial. Outcomes were accelerometer-based moderate-to-vigorous intensity physical activity (MVPA), light intensity physical activity (LiPA), standing time, and sedentary time; self-reported compliance with physical activity guidelines, body composition, physical function, and mental well-being. LSP programs included Women on Wheels/Bike for Life, Go for Life Games, Get Ireland Walking, and Men on the Move. We used a difference-in-differences approach to estimate program effects. We found evidence of positive program effects on accelerometer-derived MVPA (Women on Wheels/Bike for Life, Get Ireland Walking), LiPA (Go for Life Games), and sedentary time (Women on Wheels/Bike for Life, Go for Life Games) (*p* < .05), plus evidence of positive effects on self-reported physical activity for all LSP programs (*p* < .05). We did not find evidence of program effects on body composition. Outcomes related to physical function were mixed. Men on the Move was the only program where mental well-being scores increased significantly relative to the control group. Despite sample size limitations, the results support the effectiveness of LSP programs over a 6-month period, notably in terms of energy expenditure outcomes, while identifying areas for improvement regarding outcomes related to body composition, physical function and, particularly, mental well-being.

**Supplementary Information:**

The online version contains supplementary material available at 10.1007/s10433-025-00847-z.

## Introduction

Adults not meeting recommended physical activity (PA) guidelines, regardless of their current health status, face potential future risk of developing ill health and are, therefore, a key target for intervention seeking long-term lifestyle change (Howlett et al. 2019). Worldwide, insufficient PA is a major modifiable risk factor for noncommunicable diseases and premature mortality, creating substantial costs to the economy (Ding et al. 2016). In Ireland, only 34% of adults aged 55–65 years reported achieving National Physical Activity Guidelines and the percentage decreased to 29% in those aged 65–74 years, and 19% among those aged > 75 years (Health Service Executive 2024). Reasons why PA declines with age include higher prevalence of long-term health conditions that may make being active more challenging and uncomfortable, misconceptions and inappropriate social norms about PA in older age, and lack of access to appropriate opportunities for being physically active (e.g. facilities, services and programmes) (Health Service Executive 2024; O’Regan et al. 2020; World Health Organisation 2023).

Between 2000 and 2009, supported by public funds administered via Sport Ireland, a network of 29 local sports partnerships (LSPs) was established in Ireland to undertake a wide range of actions with the aim of increasing sport and PA participation levels in their local communities. Core investment to the LSPs has been increasing steadily, from €4.9 M in 2014 to €8.2 M in 2020 (Sport Ireland Participation 2020). Actions include providing targeted programs, events and initiatives to increase PA and sport participation. In 2020, over 340,000 people from communities across Ireland took part in sport and PA opportunities organised by LSPs. Of these, over 7000 participants took part in initiatives targeting older adults (Sport Ireland Participation 2020). These numbers, along with the strategic location of LSPs across Ireland, and the increase in recent years of core public investments to the LSPs to support their programs, events and initiatives mean that LSPs are well positioned to contribute to address the low levels of inactivity observed among middle-aged and older adults in Ireland.

In order to assess the impact of their work, LSPs have adopted the use of a single item self-report measure developed by Milton et al. (2011). As of 2020, LSPs had collected data for almost 6000 participants at registration and 3-month follow-up data for almost 1700 of these participants (Sport Ireland Participation 2020). These data reveal that LSP initiatives are reaching the intended audience, with 7 out of 10 participants not meeting the National PA Guidelines at registration and almost 20% of them being classified as inactive. Further, the data indicate that the LSP initiatives are successful in moving a significant percentage of people from inactivity towards activity over a period of 6 months. Specifically, from baseline measures to 3-month follow-up, the percentage of participants that were inactive decreased from 17 to 10% with a reduction in somewhat active participants from 54 to 49%, allowing for a substantial increase in active participants from 28 to 40% (Sport Ireland Participation 2020). The data reported, however, do not include information on the effectiveness of specific LSP programs.

Additional, and more robust, evidence about the impact of LSP programs comes from a controlled pragmatic trial of *Men on the Move*, a free, gender-sensitised, 12-week community-based ‘beginners’ PA programme for inactive adult men that uses PA as a ‘hook’ to engage men in their health with a view to improving their overall health and well-being (Carroll et al. 2019; Kelly et al. 2019). Over 900 middle-aged and older men (82% aged between 40 and 70 years), predominantly inactive and overweight/obese, took part in the evaluation. At 12 weeks, 74% of the intervention group achieved the target 1 MET increase in aerobic fitness (based on time to complete one mile), 13.5% achieved the target 5% reduction in bodyweight, and 48% achieved the target 5 cm reduction in waist circumference. At 52 weeks follow-up, 52%, 22% and 42% of the men in the intervention group were achieving the aerobic fitness, body weight and waist circumference targets, respectively. Furthermore, an economic evaluation that compared the costs (direct and indirect) of *Men on the Move* to its benefits found the program to be cost-effective in supporting participants achieve significant improvements in aerobic fitness, weight loss and waist reduction (Kelly et al. 2021).

While the available evidence is suggestive of the benefits of publicly funded LSP PA programs for inactive middle-aged and older adults, a rigorous evaluation has been conducted only in the context of one program catering specifically to men. Furthermore, the range of outcomes examined to date is still relatively narrow and does not include examination of relevant outcomes related to energy expenditure (e.g. light intensity PA, sedentary time) assessed by means of devices, such as accelerometers. Outcomes assessed to date do not include either physical function outcomes, such as functional mobility, which become increasingly important to maintain independence and autonomy as people age (Clifford et al. 2024). In addition, there is a need for pragmatic yet rigorous evaluations of real-world programs that can help us build the practice-based evidence required to adjust the compass bearing of existing policy (Ogilvie et al. 2020; Reis et al. 2016). Such evaluations appear particularly important to increase accountability in the context of programs funded with taxpayers’ money. Therefore, the aim of this study is to contribute to the existing body of evidence regarding LSP initiatives by providing evidence stemming from a controlled pragmatic trial about the effectiveness of several PA programs catering to middle-aged and older adults and expanding the range of outcomes considered.

## Methods

### Setting

This study took place in the mid-west region of Ireland. Within the LSPs, community sport and PA hubs were developed as part of Ireland’s National Physical Activity Plan with the specific focus to increase engagement in PA, generally and particularly amongst disadvantaged, marginalised and hard to reach groups. As part of the Move for Life (MFL) cluster randomised feasibility trial (Woods et al. 2024) (Clinical trial registration: https://www.isrctn.com/Registration#ISRCTN11235176), a total of eight hubs across counties Clare (*n* = 4) and Limerick (*n* = 4) were recruited. Hub inclusion criteria required professional expertise to run four nationally approved PA programs suitable for inactive middle-aged and older adults (see Table 1).

**Table 1:** Characteristics of LSP delivered physical activity programs

Program	Audience	Aim and focus	Frequency and duration	Published evidence
Women on Wheels/Bike for Life	Middle aged and older adults, catering to women only (Women on Wheels) or women and men (Bike for Life)	The aim of this program is to improve participants cycling skills, e.g. gearing/safe cycling and to increase their knowledge and confidence when cycling in groups and on the road. This program is led by trained road captains with the experience and expertise to help beginner cyclists improve	90 min once weekly for 10 weeks	No
Go for Life Games	Middle aged and older adults	The overall aim of Go For Life is to get older people more active, more often through fun and social recreational sports. Go for Life Games introduces participants to games such as Sidils—an adaptation of Ten-Pin Bowling & Skittles, and Flisk– an adaption of Frisbee & Horseshoe	90 min once weekly for 8 weeks	No
Get Ireland Walking (Active Community Walking Programme)	Middle-aged and older adults	The program is led by skilled walking facilitators and emphasises the importance and benefits (e.g. social connections) of group activity. This allows the participants to increase their PA levels, explore new areas, make new friends, and build confidence to progress to more advanced walking programs and learn new skills All walks take place on terrain that is accessible and safe, generally within and around local communities, parks, clubs and low-level woodlands	90 min once weekly for 10 weeks	No
Men on the Move	Middle-aged and older adults (men only)	The aim of this program is to use PA as a ‘hook’ to engage men in their health with a view to improving their overall health and well-being. Men on the Move is a free, gender-sensitised, community-based ‘beginners’ program for inactive adult men. Underpinned by Social Cognitive Theory, it consists of structured group exercise, two facilitated experiential workshops, a twenty-four-page health information booklet, a pedometer for independent PA sessions, weekly phone contact, a customised wallet card to record measures taken and a 5 km celebration event at the end	60 min twice weekly for 12 weeks	Yes (Carroll et al. 2019; Kelly et al. 2019, 2021)

### Trial and study design

The MFL trial used a cluster design where LSP hubs were defined as the units of randomisation (the clusters). Participants within these hubs were randomised to one of the three arms, i) the MFL intervention group (MFL; the existing PA programs plus the MFL behavioural augmentation, 3 hubs); ii) the usual provision (UP; the existing PA programs consisting of PA classes delivered as normal, 3 hubs); and iii) the control group (CON; information on PA only, 2 hubs). CON individuals were invited to participate in the PA programs once the trial was completed. Each hub was geographically separated to reduce the possibility of spill over and clusters were stratified as rural or urban (O’Regan et al. 2019; Woods et al. 2024). MFL recruited 733 individuals (May–September 2018). Due to insufficient number of available accelerometers, the intervention begun in May for participants in programs offered by the Limerick LSP and in September for participants in programs offered by the Clare LSP.

### PA programs

The four LSP programs offered to participants in the MFL trial were *Women on Wheels/Bike for Life, Go for Life Games, Get Ireland Walking,* and *Men on the Move*. In total, 32 freely accessible PA programs were implemented over the trial period. Table 1 provides details on these programs. For this study, participants in the MFL and UP arms were combined and compared to controls to ascertain if LSP PA programs catering to middle-aged and older adults work.

### Procedures

A diverse range of non-probabilistic recruitment strategies informed by our published qualitative research were used (O’Regan et al. 2020). Individuals who expressed an interest attended a ‘health check appointment’ where they were informed about the study in person, and in writing, and provided informed consent as per ethics committee approval (University of Limerick, Faculty of Education and Health Sciences Research Ethics Committee, 2018_02_15_EHS; 06 March 2018). Consenting individuals that met inclusion criteria as per the study protocol completed baseline measures and their hubs were subsequently assigned to the CON, UP or MFL arm. To be included in the trial, participants had to be inactive based on a self-report screening measure described in the study outcomes section below, community dwelling, aged 50 years and over, and able to exercise independently (i.e. ability to complete program activities with minimal or no help from others). Participants were excluded if they were aged under 50 years, active (according to the self-report screening measure), and unable to exercise independently. Outcome measures were collected at baseline (*T*0), post-intervention (*T*1, at 8, 10 or 12-weeks), and 6-month follow up (*T*2). No formal sample size calculations were conducted due to the feasibility nature of the trial (Woods et al. 2024).

### Study outcomes

#### Energy expenditure

Common to all accelerometer-based outcomes in this study, participants were required to wear the AP3M device on the anterior aspect of the right mid-thigh for 24-h/day, on eight consecutive days and were instructed to only remove the device if they were going to be submerged in water (i.e. swimming or bathing). All device removals were documented as non-wear time in a non-wear diary. To be included in all analyses for accelerometer-based data, participants were required to provide at least 4 days (3 weekdays, 1 weekend) of valid accelerometer data (≥ 10 h waking data/day) (Edwardson et al. 2017). Based on previous research in adult populations, monitor non-wear time was defined as a period of ≥ 60 min of consecutive zero-counts (Donoghue et al. 2014). The total non-wear time was summed for each day and the 24-h day adjusted accordingly.

MVPA was calculated using a previously developed and validated count-to-activity threshold (8873 counts.15 s^−1^) (Powell et al. 2017). LiPA was calculated as 24 h–[sedentary time + standing + MVPA]. Standing time and sedentary time were derived directly from the AP3M output. To differentiate sedentary time from sleep, accelerometer data were processed to identify daily time in bed (detected by a proprietary algorithm). For self-report, a clear definition of frequency, intensity, time and types of PA to meet PA guidelines PAGL was provided. In accordance with WHO guidelines (World Health Organisation 2010), self-reported compliance with PA guidelines was assessed by the number of days over a typical or usual week that participants accumulated at least 30 min of MVPA, and if 4 days or less if they had accumulated at least 2.5 h MVPA in the last 7 days. The measure used has shown acceptable properties for classifying adults as meeting PAGL (Milton et al. 2011).

#### Body composition (adiposity)

Weight and height were measured using an electronic scale (Seca model 770, Seca Ltd., Birmingham, UK) and stadiometer (Seca model 214, Seca Ltd., Birmingham, UK). Body mass index (BMI) was calculated based on the formula: weight (kg)/height (m)^2^. Waist circumference (WC) was recorded to the nearest 0.1 cm with an adjustable anthropometric un-elastic tape (Seca model 200, Seca Ltd., Birmingham, UK).

#### Physical function

Physical function was assessed by the Timed Up and Go Test (TUG) and the 6-min walk test (6MWT). TUG assesses functional mobility and balance, since it is highly correlated and concurrently valid with gait speed (Donoghue et al. 2014). From a seated position, participants were required to stand, walk 3 m, turn around, walk back and sit down, as briskly and as safely as possible. The time taken to complete the TUG test was recorded in seconds. TUG possesses high intra- and inter-rater reliability, *n* = 10–30, ICC = 0.99; ICC = 0.98 (Shumway-Cook et al. 2000). The 6MWT is a valid measure of functional exercise capacity with stable and reproducible results (Bohannon and Crouch 2017). Participants walked as briskly and safely as possible, up and down a 30 m straight flat track, continuously for 6 min. The distance that they covered was recorded to the nearest metre.

#### Psychological well-being

Psychological well-being was assessed using the seven-item version of The Warwick-Edinburgh Mental Well-being Scale (WEMWBS) (Tennant et al. 2007), which has demonstrated high correlation with other scales that measure positive mental health and well-being (Tennant et al. 2007), while being highly sensitive to changes in mental well-being (Maheswaran et al. 2012).

#### Study covariates

Questionnaires collected data on demographics (age, gender, marital status, education level, health insurance and occupational status), prevalence and type of chronic diseases (a list of 22 conditions, in accordance with the International Classification of Diseases), and on environmental conditions known to influence independent PA (perceived safety, convenience and functionality) (Franco et al. 2015).

### Data analysis

Descriptive statistics were summarised by study group at baseline and reported as means and standard deviations or *n* and percentages, as appropriate. Following inspection of distributional and missing data (missing at random) assumptions, we used generalised linear mixed models to estimate the adjusted differences in means of study outcomes between each of the LSP program groups and CON at each time point and explore differences in patterns of change over time while accounting for correlation introduced by repeated measurements. Consistent with a difference-in-differences approach to estimate intervention/program effects, we sought to determine whether differences between groups are different at different occasions by testing for group-by-time interaction effects (Leppink et al. 2017; Malesky et al. 2014).

Based on an ecological perspective of active living (Franco et al. 2015), a comprehensive set of covariates was considered (Online Resource 1) and each covariate examined to understand how they relate independently to the initial status (i.e. starting value) and rate of linear change of the outcomes. The LSP (Clare, Limerick), by which the randomisation was stratified, was accommodated by its inclusion as a covariate in the model. We controlled for the effects of the MFL trial by including the difference between the MFL (experimental) group and the usual provision (‘intact’ LSP programs) group as another covariate in the models.

A categorical variable ‘group’ was tested to explore any differences in the initial status and changes over time (i.e. interaction with time) between each of the LSP programs considered and the CON group. We accounted for the hierarchical structure of the data by clustering observations within participants as a random effect. For each outcome, variables with p values > 0.10 in the initial models, and variables central to the research questions (e.g. group, time, and their interaction, LSP), were included in a subsequent multivariable model. We tested several covariance structures appropriate for longitudinal data (unstructured, compound symmetry, first-order autoregressive) to determine the error covariance structure that best fit the data.

The models for continuous outcome variables were calculated using the Generalised Linear Mixed Models procedure with maximum likelihood estimation in SPSS version 29, whereas the model for the categorical outcome variable ‘compliance with PAGL’ was calculated using the Generalised Estimating Equations procedure in SPSS version 29. We used all available observations to obtain maximum likelihood estimates and fit the models to observed data.

Differences in adjusted means at each time point are presented with their corresponding 95% confidence intervals and p-values, which are considered mainly exploratory due to the lack of formal sample size calculations. Consistent with previous work using a difference-in-differences approach to estimate the effects of interventions (Malesky et al. 2014), and the predominantly exploratory nature of this study, we used a 90% confidence level to ascertain the statistical significance of group-by-time interactions. This was also deemed necessary due to low sample size in some of the study groups and the resulting reduction in statistical power to detect interactions (Brysbaert 2019).

## Results

Of 733 recruited individuals, 98% (*n* = 724) consented and completed baseline measures. Of those, 18% (*n* = 132) did not meet the eligibility criteria and were excluded due to age (less than 50 years) or activity status (meet PA guidelines), even though the individuals whose data were excluded for the latter circumstance considered themselves ‘inactive’ (according to the guidelines) upon recruitment. Individuals who were excluded for not meeting eligibility criteria were younger (59.4 vs 63.06, *p* < .001) and more active (activPAL MVPA mins (10 min bouts) 32.12 vs 13.02, *p* < .001), and with a higher percentage of males compared to participants who met eligibility criteria (29.9% vs 19.6%, *p* < .01). An additional 133 individuals who met eligibility criteria and attended all or some of the scheduled assessments but reportedly did not attend a program were further excluded from the analyses.

Table 2 presents the baseline characteristics of the 468 participants included in the analyses. A majority were female (79.7%), 45.6% were living with three or more chronic conditions, and most (80.5%) were overweight or obese. On average, participants in the CON group were younger than in Go for Life Games and older than Women on Wheels/Bike for Life participants, while the proportion of males and females in the CON group also differed from what was observed in Get Ireland Walking and Men on the Move (*p* < .05).

**Table 2:** Baseline (*T*0) characteristics of study participants by LSP program (*N* = 468)

Descriptive variables	LSP program				
	WOW/BFL	GFL	GIW	MOM	CON
Age: mean (SD), *n*	59.3 (7.3), 42	67.5 (7.8), 67	63.2 (7.5), 192	63.1 (8.1), 24	62.5 (7.3), 143
Gender: *n* (%)					
Male	7 (16.7)	10 (14.9)	23 (12.0)	24 (100.0)	31 (21.7)
Female	35 (83.3)	57 (85.1)	169 (88.0)	–	112 (78.3)
Level of education: *n* (%)					
Primary or no formal training	2 (5.0)	12 (18.5)	9 (4.8)	4 (16.7)	6 (4.3)
Lower secondary	8 (20.0)	14 (21.5)	21 (11.2)	3 (12.5)	23 (16.3)
Upper secondary	8 (20.0)	12 (18.5)	60 (32.1)	4 (16.7)	32 (22.7)
Post-secondary, non-tertiary	1 (2.5)	5 (7.7)	9 (4.8)	1 (4.2)	3 (2.1)
Non degree	9 (22.5)	12 (18.5)	52 (27.8)	2 (8.3)	44 (31.2)
Degree or higher	12 (30.0)	10 (15.4)	36 (19.3)	10 (41.7)	33 (23.4)
Medical card: *n* (%)					
Yes	10 (25.6)	36 (56.3)	53 (28.5)	10 (41.7)	45 (31.9)
No	29 (74.4)	28 (43.8)	133 (71.5)	14 (58.3)	96 (68.1)
Marital status: *n* (%)					
Married/living with partner	29 (72.5)	35 (53.8)	126 (67.0)	24 (100.0)	87 (61.7)
Other	11 (27.5)	30 (46.2)	62 (33.0)	–	54 (38.3)
BMI category: *n* (%)					
Underweight	–	1 (1.5)	2 (1.1)	–	–
Normal	7 (16.7)	6 (9.1)	46 (24.3)	1 (4.5)	27 (19.1)
Overweight	15 (35.7)	25 (37.9)	61 (32.3)	11 (50.0)	65 (46.1)
Obese	20 (47.6)	34 (51.5)	80 (42.3)	10 (45.5)	49 (34.8)
Number of chronic health conditions: *n* (%)					
Less than three	26 (76.5%)	22 (40.7%)	84 (53.2%)	10 (52.6%)	57 (56.4%)
Three or more	8 (23.5%)	32 (59.3%)	74 (46.8%)	9 (47.4%)	44 (43.6%)
Area deprivation index: *n* (%)					
Marginally below average	27, (64.3)	54 (80.6)	164 (85.4)	18 (75.0)	49 (43.3)
Disadvantaged	15 (35.7)	13 (19.4)	28 (14.6)	6 (25.0)	94 (65.7)
Geographical location: *n* (%)					
Rural	21 (50.0)	20 (29.9)	83 (43.2)	16 (66.7)	–
Urban	21 (50.0)	47 (70.1)	109 (56.8)	8 (33.3)	143 (100.0)
MVPA mins (10 min bouts) (SD), *n*	11.01 (13.55), 34	11.39 (13.54), 55	13.13 (12.74), 144	15.72 (13.84), 17	13.05 (12.87), 104
Sedentary time (h) (SD), *n*	9.22 (1.78), 34	9.39 (1.87), 55	8.80 (1.57), 144	8.83 (1.60), 17	8.96 (1.66), 104

WOW/BFL, Women on wheels/bike for life; GFL, Go for life games; GIW, Get Ireland walking; MOM, Men on the move; CON, Control; BMI, Body mass index; MVPA mins (10 min bouts), MVPA minutes/day (in bouts of 10 min) (lower Ns for MVPA and sedentary time than in other variables reflect the circumstance that accelerometers were worn by a subsample of participants)

The overall retention rate for the MFL trial was 63%. The number of participants for this specific study at each time point and the unadjusted means of the primary and secondary outcomes and are shown in Online Resource 2, while Table 3 displays the difference-in-differences analysis examining whether changes in study outcomes over time (*T*0, *T*1, *T*2) vary as a function of LSP program. Table 4 shows the percentage change in adjusted means of the study outcomes for each group.

**Table 3:** Difference-in-differences estimation of changes in outcome variables

	Adjusted mean difference *T*0	*p* value	Adjusted mean difference *T*1	*p* value	Adjusted mean difference *T*2	*p* value	Group × time	*p* value
PAGL (%)								
WOW/BFL versus CON	− 3.0 (− 22.0, 16.0)	0.757	17.0 (2.0, 31.0)	0.024	25.0 (10.0, 40.0)	0.001	2.93 (1.64, 5.23)	.002^†^
GFL versus CON	− 20.0 (− 37.0, − 2.0)	0.03	11.0 (− 5.0, 27.0)	0.181	8.0 (− 11.0, 27.0)	0.405	1.94 (1.26, 3.00)	.012^†^
GIW versus CON	− 5.0 (− 18.0, 9.0)	0.497	10.0 (− 2.0, 23.0)	0.111	17.0 (4.0, 31.0)	0.012	1.81 (1.31, 2.51)	.003^†^
MoM versus CON	− 26.0 (− 51.0, − 1.0)	0.044	7.0 (− 17.0, 31.0)	0.563	11.0 (− 14.0, 37.0)	0.381	2.32 (1.23, 4.37)	.030^†^
MVPA (min)								
WOW/BFL versus CON	− 1.16 (− 10.2, 7.88)	0.801	8.91 (− 1.58, 19.40)	0.096	11.15 (1.34, 20.95)	0.026	5.75 (2.26, 9.24)	.007^†^
GFL versus CON	− 1.33 (− 9.33, 6.66)	0.743	− 0.78 (− 9.71, 8.16)	0.864	5.17 (− 4.40, 14.73)	0.288	1.75 (− 1.54, 5.04)	0.38
GIW versus CON	− 1.92 (− 8.16, 4.31)	0.544	7.40 (0.31, 14.50)	0.041	6.87 (− 0.08, 13.81)	0.053	3.92 (1.49, 6.36)	.008^†^
MoM versus CON	− 4.22 (− 16.7, 8.21)	0.505	− 3.13 (− 15.92, 9.65)	0.63	− 0.70 (− 14.51, 13.11)	0.92	1.13 (− 3.32, 5.58)	0.675
LiPA (h)								
WOW/BFL versus CON	0.07 (− 0.14, 0.28)	0.521	0.09 (− 0.14, 0.33)	0.44	0.19 (− 0.03, 0.42)	0.095	0.04 (− 0.02, 0.10)	0.251
GFL versus CON	0.01 (− 0.20, 0.21)	0.94	0.05 (− 0.18, 0.27)	0.685	0.27 (0.03, 0.51)	0.031	0.07 (0.01, 0.13)	.078^†^
GIW versus CON	− 0.03 (− 0.18, 0.12)	0.726	0.01 (− 0.16, 0.18)	0.902	0.01 (− 0.16, 0.18)	0.909	0.01 (− 0.03, 0.06)	0.699
MoM versus CON	0.00 (− 0.29, 0.29)	0.988	0.03 (− 0.27, 0.33)	0.832	− 0.05 (− 0.38, 0.28)	0.759	− 0.01 (− 0.09, 0.07)	0.764
Stand (h)								
WOW/BFL versus CON	− 0.12 (− 0.72, 0.48)	0.694	0.12 (− 0.65, 0.89)	0.759	0.05 (− 0.72, 0.82)	0.9	0.08 (− 0.14, 0.30)	0.545
GFL versus CON	− 0.07 (− 0.65, 0.51)	0.809	− 0.50 (− 1.22, 0.22)	0.172	− 0.57 (− 1.37, 0.24)	0.165	− 0.16 (− 0.39, 0.08)	0.266
GIW versus CON	0.34 (− 0.08, 0.77)	0.114	− 0.27 (− 0.82, 0.28)	0.333	− 0.26 (− 0.83, 0.31)	0.367	− 0.24 (− 0.40, − 0.07)	.018^†^
MoM versus CON	0.60 (− 0.23, 1.43)	0.156	0.26 (− 0.70, 1.21)	0.6	0.18 (− 0.90, 1.26)	0.743	− 0.08 (− 0.37, 0.21)	0.638
Sed. time (h)								
WOW/BFL versus CON	0.29 (− 0.44, 1.02)	0.428	− 0.64 (− 1.48, 0.20)	0.136	− 0.71 (− 1.54, 0.12)	0.093	− 0.44 (− 0.74, − 0.14)	.017^†^
GFL versus CON	0.77 (0.12, 1.43)	0.021	.022 (− 0.73, 0.77)	0.953	− 0.41 (− 1.24, 0.42)	0.328	− 0.55 (− 0.84, − 0.26)	.002^†^
GIW versus CON	0.21 (− 0.30, 0.72)	0.418	0.24 (− 0.35, 0.83)	0.427	− 0.09 (− 0.68, 0.51)	0.779	− 0.17 (− 0.38, 0.05)	0.208
MoM versus CON	− 0.20 (− 1.23, 0.83)	0.705	0.02 (− 1.05, 1.09)	0.967	0.18 (− 1.00, 1.37)	0.763	.078 (− 0.32, 0.47)	0.746
BMI								
WOW/BFL versus CON	0.52 (− 0.57, 1.61)	0.352	0.57 (− 0.72, 1.86)	0.387	0.78 (− 9.49, 2.04)	0.228	0.11 (− 0.21, 0.42)	0.574
GFL versus CON	− 0.17 (− 1.21, 0.87)	0.745	− 0.48 (− 1.70, 0.73)	0.435	− 0.57 (− 1.77, 0.64)	0.353	0.08 (− 0.21, 0.38)	0.648
GIW versus CON	− .020 (− 0.94, 0.54)	0.596	− 0.89 (− 1.78, − 0.00)	0.049	− 0.83 (− 1.65, − 0.00)	0.05	− 0.12 (− 0.33, 0.09)	0.33
MoM versus CON	− 0.12 (− 1.52, 1.27	0.861	− 0.11 (− 1.71, 1.50)	0.894	0.76 (− 0.77, 2.29)	0.328	0.28 (− 0.11, 0.66)	0.239
Waist Circ. (cm)								
WOW/BFL versus CON	− 0.27 (− 2.90, 2.37)	0.842	− 1.35 (− 4.51, 1.82)	0.403	− 0.98 (− 3.96, 1.99)	0.516	− 0.40 (− 1.47, 0.64)	0.535
GFL versus CON	1.05 (− 1.47, 3.58)	0.412	2.07 (− 0.92, 5.06)	0.174	1.16 (− 1.69, 4.00)	0.425	− 0.46 (− 1.44, 0.51)	0.441
GIW versus CON	0.90 (− 0.96, 2.75)	0.341	2.82 (0.57, 5.07)	0.014	2.15 (0.10, 4.21)	0.04	0.23 (− 0.50, 0.96)	0.611
MoM versus CON	0.76 (− 2.69, 4.20)	0.666	0.72 (− 3.32, 4.76)	0.726	− 1.72 (− 5.42, 1.99)	0.363	− 1.27 (− 2.63, 0.05)	0.118
TUG (s)								
WOW/BFL versus CON	0.22 (− 0.28, 0.72)	0.389	0.24 (− 0.33, 0.81)	0.406	− 0.18 (− 1.10, 0.72)	0.685	− 0.23 (− 0.45, − 0.01)	.094^†^
GFL versus CON	0.35 (− 0.13, 0.83)	0.148	1.24 (0.69, 1.79)	< .001	1.36 (0.53, 2.20)	0.001	0.21 (0.02, 0.40)	.074^†^
GIW versus CON	− 0.07 (− 0.41, 0.27)	0.69	0.64 (0.25, 1.04)	0.002	0.65 (0.07, 1.23)	0.028	0.18 (0.04, 0.32)	.039^†^
MoM versus CON	− 0.08 (− 0.75, 0.58)	0.804	− 0.24 (− 0.98, 0.51)	0.535	− 0.07 (− 1.14, 1.00)	0.899	− 0.10 (− 0.36, 0.16)	0.523
6MWT (m)								
WOW/BFL versus CON	− 32.9 (− 63.8, − 1.99)	0.037	25.1 (− 4.6, 54.8)	0.097	17.6 (− 18.8, 53.9)	0.341	26.0 (13.4, 38.7)	< .001^†^
GFL versus CON	− 44.0 (− 73.9, − 14.1)	0.004	− 27.7 (− 60.5, 4.99)	0.096	− 32.9 (− 70.5, 4.56)	0.085	7.91 (− 5.11, 20.9)	0.317
GIW versus CON	− 15.0 (− 36.6, 6.6)	0.173	− 5.72 (− 26.8, 15.3)	0.593	10.7 (− 14.3, 35.8)	0.4	15.1 (6.59, 23.6)	.004^†^
MoM versus CON	− 17.3 (− 57.9, 23.4)	0.404	15.8 (− 22.1, 53.6)	0.413	− 12.9 (− 57.6, 31.7)	0.568	6.96 (− 8.78, 22.7)	0.466
Well-being								
WOW/BFL versus CON	0.10 (− 1.55, 1.76)	0.904	− 0.28 (− 2.02, 1.46)	0.748	− 0.01 (− 1.89, 1.86)	0.99	− 0.30 (− 1.02, 0.43)	0.501
GFL versus CON	0.76 (− 0.79, 2.31)	0.337	− 1.18 (− 2.85, 0.49)	0.166	− 0.89 (− 2.56, 0.78)	0.296	− 0.57 (− 1.21, 0.08)	0.146
GIW versus CON	0.10 (− 1.03, 1.24)	0.858	− 1.09 (− 2.32, 0.14)	0.081	− 0.48 (− 1.68, 0.71)	0.426	− 0.03 (− 0.50, 0.44)	0.914
MoM versus CON	− 1.93 (− 4.10, 0.24)	0.081	− 2.08 (− 4.36, 0.19)	0.072	− 0.47 (− 2.67, 1.74)	0.677	0.91 (0.03, 1.80)	.089^†^

WOW/BFL, Women on wheels/bike for life; GFL, Go for life games; GIW, Get Ireland walking; MOM, Men on the move; CON, Control; MVPA, Moderate to vigorous physical activity; LiPA, Light physical activity; Stand, Time standing; Sed. Time, Sedentary time during waking hours; PAGL, Compliance with physical activity guidelines; BMI, Body mass index; Waist Circ., Waist circumference; TUG, Timed up and go test; 6MWT, 6-Minute walk test; Well-being, Mental well-being

^†^significant group-by-time interaction indicative of program effects

**Table 4:** Percentage change in outcome variables according to study group (*T*0–*T*2)

	WOW/BFL (%)	GFL (%)	GIW (%)	MOM (%)	CON (%)
PAGL	58.6	82.9	50.0	122.9	9.8
MVPA	− 1.5	− 18.2	− 11.9	− 29.2	− 35.6
LiPA	2.1	9.6	− 4.6	− 11.9	− 8.2
Stand	11	− 4.7	− 6.5	− 2.7	6.8
Sed. Time	− 5.8	− 7.4	1.9	9.8	5.3
BMI	− 0.3	− 2.5	− 3.3	1.9	− 1.1
Waist circ	− 4.1	− 3.2	− 2.1	− 5.8	− 3.4
TUG	− 12.9	6.6	2.9	− 7.4	− 7.5
6MWT	14.4	4.3	8.6	4	3.8
Well-being	1.9	− 4.2	0	8.9	2.3

Percentage changes are based on adjusted means; WOW/BFL, Women on wheels/bike for life; GFL, Go for life games; GIW, Get Ireland walking; MOM, Men on the move; CON, Control; MVPA, Moderate to vigorous physical activity; LiPA, Light physical activity; Stand, Time standing; Sed. Time, Sedentary time during waking hours; PAGL, Compliance with physical activity guidelines; BMI, Body mass index; Waist Circ., Waist circumference; TUG, Timed up and go test; 6MWT, 6-Minute walk test; Well-being, Mental well-being

### Difference-in-differences analysis

As seen in Table 3, when looking at device-based energy expenditure outcomes in the study subsample that wore accelerometers (*n* = 376), we observed significant group-by-time interactions favouring *Women on Wheels/Bike for Life* and *Get Ireland Walking* over CON in daily minutes of MVPA. The percentage change in adjusted means indicate that MVPA decreased over time in all study groups, but notably less so in *Women on Wheels/Bike for Life* (− 1.5%) and in *Get Ireland Walking* (− 11.9%) than in CON (− 35.6%). The interaction term comparing *Go for Life Games* and CON regarding daily hours of LiPA was also significant, which corresponds to an average increase of 9.6% in the former and a decrease of 8.2% in the latter. Contrary to expectations, the significant interaction comparing *Get Ireland Walking* and CON participants for standing time revealed that while the latter increased the number of hours they spent standing daily (6.8%), the former experienced a decrease (− 6.5%). Lastly, significant interactions showed that while CON participants increased the time they spent being sedentary over the 6-month study period (5.3%), participants in the *Go for Life Games* and *Women on Wheels/Bike for Life* groups engaged less in such behaviour over time (− 7.4% and − 5.8%, respectively).

When considering the percentage of participants who reported meeting the PA guidelines, all group-by-time interactions comparing LSP programme groups with the CON group were statistically significant. In terms of percentage change in adjusted proportions, all groups experienced increases, but these were substantially larger in *Men on the Move* (122.9%), *Go for Life Games* (82.9%), *Women on Wheels/Bike for Life* (58.6%), and *Get Ireland Walking* (50.0%) than in CON (9.8%).

We did not find significant interactions for any comparisons involving LSP programme groups and CON for the body composition outcomes examined in this study (BMI, waist circumference). This suggests that the trajectories of these outcomes over time were largely independent of the study groups considered.

Regarding physical function outcomes, the significant interactions comparing *Women on Wheels/Bike for Life*, *Go for Life Games* and *Get Ireland Walking*, respectively, with CON in the TUG revealed a mixed pattern of findings. Specifically, while the amount of time needed to complete the test decreased more among *Women on Wheels/Bike for Life* participants (− 12.9%) than among CON participants (− 7.5%), it did increase, unexpectedly, among *Go for Life Games* (6.6%) and, to a lesser extent, *Get Ireland Walking* participants (2.9%). On the other hand, both *Women on Wheels/Bike for Life* and *Get Ireland Walking* participants outperformed their CON counterparts in the number of metres completed in 6MWT (increases of 14.4% and 8.6% vs 3.8%, respectively), and the differences can be considered statistically significant as per the interactions shown in Table 3.

Finally, the pattern of group-by-time interactions for comparisons involving LSP programme groups and CON for psychological well-being scores suggests, as in the case of body composition outcomes, that the rate of change of well-being was independent of the study groups considered, with the notable exception of *Men on the Move*. As shown in Table 4, the average increase in well-being among *Men on the Move* participants (8.9%) was noticeably larger than among CON participants (2.3%) and the *p* value of the corresponding interaction term was statistically significant at the specified confidence level (Table 3).

## Discussion

In response to calls for more evaluations of real-world programs in public health (Gertler et al. 2016), this study presents a pragmatic, yet rigorous, evaluation of the effectiveness of state funded LSP PA programs for middle-aged and older adults in Ireland. Accelerometer-derived PA provided evidence of positive program effects. Cycling programs were shown to improve MVPA and decrease sedentary time relative to controls. For walking programs, similarly, MVPA was positively impacted, while a structured games program increased LiPA and decreased sedentary time in the oldest group recruited. In addition, we found evidence of positive effects on self-reported compliance with PA guidelines for all four programs. We did not find evidence of program effects on body composition. Regarding physical function, while we observed a mixed pattern of findings in the TUG, similar to MVPA we documented positive program effects for participants in cycling and walking programs in the 6MWT. Lastly, *Men on the Move* was the only program where we observed a significant improvement in mental well-being scores relative to controls.

The differences concerning accelerometer-derived PA seem logical considering the nature of the cycling and walking programs studied, which encourage continuous activity of at least moderate intensity (e.g. brisk walking), and the more intermittent, lighter intensity activity that results from the friendly competition and cooperative games that characterise the *Go for Life Games* program. Of note, as shown in Table 4, accelerometer-derived MVPA declined over the 6-month period in all groups considered in this study, a finding consistent with suggestions that PA generally declines over time in adults and older adults (Health Service Executive 2024). Consequently, the superior performance of the cycling and walking programs relative to the CON group noted above signifies a protective effect against declines in MVPA observed in the overall sample over the duration of the study.

Participants in all the LSP programs analysed in this study reported substantial increases in compliance with PA guidelines when compared to the CON group over the period considered. These results are consistent with a previous evaluation of LSP programs using self-reported PA (Sport Ireland Participation 2020). While positive, the results are not necessarily surprising considering that many of the participants in these programs (as in the case of this study) are physically inactive at baseline. Therefore, attending a PA program one or 2 days a week is likely to make a noticeable difference in the activity patterns of these participants. Perhaps more encouraging is that the large differences in reported compliance with PA documented in this study for all LSP programs relative to the CON group took place over a period of 6-months that includes 3-month follow-up post-intervention. This signals potential for maintenance of program effects at the individual level, and stands in apparent contrast with a review concluding there is insufficient evidence of long-term improvements stemming from PA programs for community dwelling older adults (Olanrewaju et al. 2016). It also differs from suggestions that after 20 weeks following a programme PA behaviour returns near to baseline levels in previously inactive adults (Gomersall et al. 2015).

Contrary to initial expectations, participants in Get Ireland Walking experienced a reduction in the number of hours they spent standing daily, and the difference with participants in the CON group was statistically significant. Although standing time is different from, and preferable to, sedentary time, this finding resonates with previous research showing that PA interventions are often ineffective at reducing sedentary time (Prince et al. 2014; Martin et al. 2015). For example, PA interventions may substitute sitting time for standing time when people are active, but this does not necessarily address the issue of prolonged sedentary behaviour, which in Ireland affects all age groups, and particularly those aged 75 and older on weekends (Health Service Executive 2024). Having said this, when compared to CON, over the 6-month study period we observed significant decreases in sedentary time in both WOW/BFL and GFL programme participants, but a similar pattern was not apparent among participants in the remaining LSP programmes, particularly MOM. Furthermore, although encouraging, the observed decrease in sedentary behaviour for WOW/BFL and GFL programme participants fell short of the minimal clinically important difference (MCID) for cardiovascular disease risk reduction (Fenton et al. 2017). Once again, this is indication of the deeply ingrained nature of sedentary behaviour and illustrates the difficulty of maintaining meaningful change in middle aged and older adults, particularly with PA interventions only.

We did not find significant differences over time between LSP programs and CON neither in BMI nor in waist circumference, the two indicators of body composition (adiposity) assessed in this study. This contrasts with a previous analysis of the MFL dataset comparing the MFL intervention group (regular LSP programs + behavioural augmentation), the Usual Provision (regular LSP programs) and the CON (information on PA only) groups (Woods et al. 2024). Specifically, this analysis showed that waist circumference declined significantly in the MFL intervention group relative to both the Usual Provision and the CON groups over the 6-month study period. Similarly, self-reported compliance with PA guidelines increased significantly in MFL relative to Usual Provision and CON and in Usual Provision relative to CON. This suggests that for changes in indicators of adiposity to occur, in combination with changes in PA, enriching regular LSP PA programs with behaviour change strategies such as the ones used in the MFL intervention (Bengoechea et al. 2021) may be necessary. These strategies aimed to: (1) help participants develop cognitive and behavioural skills to regulate their own PA behaviour (e.g. goal setting and self-monitoring, relapse prevention), (2) offer opportunities to socialise, provide and get support, and develop feelings of connectedness to others and belonging, and (3) build group cohesion by creating positive group dynamics and fostering a sense of group identity in relation to norms for participation in PA. The emphasis of the MFL intervention on promoting social connectedness echoes research that shows the critical importance for health and well-being of maintaining strong social bonds as people age (Holt-Lunstad et al. 2015). The findings from the current study, in combination with results from a previous evaluation of the Men on the Move program (Carroll et al. 2019; Kelly et al. 2019), the only LSP program we examined that was offered on a 2 sessions/week basis, suggest as well that a higher program dose may be necessary for changes in body composition outcomes to occur.

The findings concerning the measures of physical function (TUG, 6MWT) reveal as well some room for improvement in LSP programs when judged against other relevant interventions, particularly regarding the former measure. While participants in the cycling programs improved their performance in the TUG relative to controls, the performance of *Go for Life Games* and Get Ireland Walking participants, unexpectedly, worsened over time relative to controls. While these inconsistent findings concerning functional mobility are in line with a systematic review of exercise interventions in community-dwelling frail older adults (Giné-Garriga et al. 2014), in a recent study seeking to provide preliminary evidence for the Music and Movement for Health (MMH) programme among community-dwelling older adults in Ireland, the intervention group was found to perform better than the control group in several physical function tests, including the TUG (Clifford et al. 2024). One of the main activities comprising MMH, dancing, can contribute to improved physical capacities such as functional mobility and balance (Clifford et al. 2024), which can help explain the differences noted with our evaluation. Given the importance of functional mobility and balance for healthy ageing, LSP programs might benefit from incorporating a greater focus on such dimensions of physical performance as part of their regular activities. Similar to participants in the cycling programs, though, *Get Ireland Walking* participants outperformed, as expected, controls in the 6MWT.

Consistent with what we observed for adiposity indicators of body composition, with the notable exception of *Men on the Move*, the trajectories of mental well-being over time revealed no program effects compared to the CON group. Although not using the same measure of well-being, this finding contrast again with results from the MMH programme. It must be noted, however, that outcomes in MMH were assessed at baseline and after 12 weeks (i.e. there was no follow up period post-intervention). Furthermore, similar to another study reporting improved subjective well-being among inactive older adults partaking in a PA intervention (Stathi et al. 2020), MMH groups were smaller, met more often and had a higher staff to participant ratio, which may explain some of the differences observed with LSP programs in the current study.

The findings concerning the positive well-being effects of *Men on the Move* corroborate previous findings from a rigorous mixed methods evaluation of the program undertaken across several counties in Ireland (Carroll et al. 2019; Kelly et al. 2019). In both studies, quantitative changes in mental well-being, although significant in relation to a control group, were commensurable and relatively small. However, it was evident from the qualitative interviews in the mixed methods evaluation study that *Men on the Move* provided participants with an opportunity to make connections that acted as a powerful catalyst for change in their lives (Carroll et al. 2019). Assuming that it may not always be feasible for LSPs to increase the duration and/or frequency of other PA programs for middle aged and older adults, findings from the MFL trial suggests ways of improving the psychosocial outcomes of these programs as currently scheduled. Notably, in this trial, participants who received the behavioural augmentation mentioned earlier (MFL intervention group) increased significantly their levels of mental well-being compared to controls, with no differences observed between participants in LSP regular programs (UP) and controls (Woods et al. 2024).

### Limitations and strengths

The small sample size in some of the study groups may have constrained our ability to detect statistically significant differences when comparing each of the LSP programs to the control group. Caution is advised especially when interpreting the findings concerning *Men on the Move*. Nevertheless, this is the only program for which a robust evaluation already exists, and our pattern of results is consistent overall with the findings reported in this evaluation. It is also possible that the statistical procedures we used to control for the effects of the MFL behavioural augmentation did not fully account for the effects of this intervention augmentation in the differences reported. However, the patterns we observed in this study for the LSP programs are mostly consistent with what was observed in the UP group when compared to the MFL intervention group in the evaluation of the MFL trial (Woods et al. 2024), suggesting that the statistical adjustment worked as intended. Similar considerations apply to the temporally staggered nature of the intervention, which may have introduced an element of seasonality, despite accounting for LSP (Limerick, Clare) in the models examining program effects. Moreover, nearly 80% of study participants were women, which does not match the usually more balanced gender breakdown of LSP initiatives (Sport Ireland Participation 2020). Another limitation concerns the self-reported nature of compliance with PA guidelines. Although we used a validated self-report measure, which is consistent with the measure used by Sport Ireland to evaluate the impact of LSP programs and the Health Service Executive to monitor levels of PA in Ireland, this measure is prone to biases in terms of over-estimation and under-estimation. Therefore, we used this measure in combination with device-based assessment of PA, which also has strengths and limitations, to obtain a more nuanced and balanced picture of the PA behaviour of the participants in the study.

This study has also important strengths. Notably, it provides data on a wider range of outcomes than those examined previously (Carroll et al. 2019; Kelly et al. 2019; Sport Ireland Participation 2020). Likewise, compared to previous evaluations of LSP programs, the statistical analysis takes into consideration a much larger number of potential confounders. In addition, unlike some of the existing evaluations (Sport Ireland Participation 2020), this evaluation includes not only a control group, but also one that is comparable following rigorous randomisation as part of a larger trial.

## Conclusions and future directions

In response to calls for more evaluations of real-world programs in public health, this study presents a pragmatic, yet rigorous, evaluation of the effectiveness of state funded LSP PA programs for middle-aged and older adults in Ireland. Overall, the findings indicate that these programs are effective in improving energy expenditure outcomes over a 6-month period, particularly regarding self-reported PA. In addition, the results suggest there is room for improvement regarding the potential of some of these programs to have an effect on outcomes related to accelerometer-derived PA, body composition, physical function, and mental well-being. For this potential to be realised, a larger program dose (e.g. increased duration and/or frequency) and/or appropriate behavioural enrichment may be necessary in some cases. Evaluations using larger samples for all programs considered and longer follow-up periods are needed to gain a more accurate and complete picture of the impact of LSP PA programs for middle aged and older adults in Ireland. Likewise, evaluations incorporating qualitative data on the participants’ experience as part of a mixed-methods research design are more likely to help us understand the processes and mechanisms at stake in the differences observed. Lastly, economic evaluations will make an important contribution to our understanding of the cost-effectiveness of LSP programs and, more generally, of PA programs supported by public funds.

## Supplementary Information

Below is the link to the electronic supplementary material.

Supplementary file1 (DOCX 35 KB)

Supplementary file2 (DOCX 54 KB)

Supplementary file3 (DOCX 61 KB)

## Data Availability

In compliance with ethics approval requirements, the data underlying the results presented in the study are available from the authors upon reasonable request.
