# Supplementary material for: Do publicly funded community physical activity programs for middle-aged and older adults in Ireland work?
Source: Eur J Ageing. 2025 Mar 18;22(1):10. doi: 10.1007/s10433-025-00847-z (PMC11920450; doi:10.1007/s10433-025-00847-z)
Supplement: Supplementary file 2 — Supplementary file2 (DOCX 54 KB) [file 10433_2025_847_MOESM2_ESM.docx]

*Unadjusted values of outcome variables across the study groups at baseline (T0) and time 2 (T2) (mean (SD); n)*

|  | **Time Point 0 – Study Group** | | |  |  | **Time Point 2 – Study Group** | | |  |  |
| --- | --- | --- | --- | --- | --- | --- | --- | --- | --- | --- |
|  | **WOW/BFL** | **GFL** | **GIW** | **MOM** | **CON** | **WOW/BFL** | **GFL** | **GIW** | **MOM** | **CON** |
| MVPA  (min.) | 32.39 (20.13);  34 | 26.56 (22.07); 55 | 33.34 (18.12); 144 | 40.16 (25.03); 17 | 33.54 (19.11); 104 | 30.68 (18.34); 21 | 19.37 (21.76); 31 | 29.27 (18.21); 90 | 34.68 (19.11); 11 | 25.32 (15.45); 66 |
| LiPA  (hours) | 1.42 (0.45); 34 | 1.31 (0.51); 55 | 1.44 (0.45); 144 | 1.35 (0.41); 17 | 1.41 (0.48); 104 | 1.43 (0.46); 21 | 1.42 (0.58); 31 | 1.35 (0.37); 90 | 1.17 (0.31); 11 | 1.26 (0.38); 66 |
| Stand  (hours) | 4.38 (1.41); 34 | 4.26 (1.55); 55 | 4.97 (1.23); 144 | 4.65 (1.52); 17 | 4.56 (1.32); 104 | 4.65 (1.59); 21 | 4.59 (2.73); 31 | 4.66 (1.15); 90 | 4.61 (1.56); 11 | 4.40 (1.28); 66 |
| Sed. Time (hours) | 9.22 (1.79); 34 | 9.39 (1.87); 55 | 8.81 (1.58); 144 | 8.83 (1.60); 17 | 8.97 (1.67); 104 | 9.01 (1.63); 21 | 8.90 (1.66); 31 | 8.97 (1.43); 90 | 9.41 (1.50); 11 | 9.43 (1.45); 66 |
| PAGL (%; n) |  |  |  |  |  |  |  |  |  |  |
| *Active* | 46.3;  19 | 40.3; 27 | 42.9; 81 | 40.9; 9 | 51.4; 72 | 87.0; 20 | 68.1; 32 | 80.6; 108 | 76.5; 13 | 62.9; 61 |
| *Insufficient* | 53.7; 22 | 59.7; 40 | 57.1; 108 | 59.1; 13 | 48.6; 68 | 13.0; 3 | 31.9; 15 | 19.4; 26 | 23.5; 4 | 37.1; 36 |
| BMI | 30.13 (6.55); 42 | 30.26 (5.42); 66 | 29.03; 5.49 (192) | 29.86 (3.58); 23 | 28.91 (5.50); 142 | 31.17 (5.94); 24 | 30.03 (5.52); 48 | 28.46 (5.40); 140 | 30.35 (3.71); 17 | 28.88 (5.35); 98 |
| Waist Circ.  (cm.) | 97.24 (15.89); 42 | 98.97 (15.67); 67 | 95.19; 14.19 (192) | 105.93 (8.94); 23 | 94.78 (14.22); 141 | 95.09 (14.34); 24 | 96.48 (16.67); 48 | 90.51 (13.73); 140 | 103.39 (9.63); 17 | 91.59 (15.00); 98 |
| TUG  (sec.) | 6.75 (1.89); 42 | 7.91 (2.55); 67 | 7.09 (2.05); 192 | 6.49 (1.33); 23 | 6.49 (1.17); 142 | 5.96 (1.09); 24 | 8.31 (3.30); 48 | 6.71 (2.05); 140 | 6.00 (1.04); 17 | 6.06 (1.06); 98 |
| 6MWT  (meters) | 551.09 (69.74); 40 | 494.98 (103.59); 63 | 540.12 (87.08);  188 | 567.17(72.40); 23 | 568.54 (73.25); 142 | 612.91; (68.16); 22 | 513.94 (122.76); 36 | 594.25 (87.75) 127 | 610.06 (63.61); 16 | 585.72 (79.36); 88 |
| Wellbeing  (Score: 7-35) | 24.91 (5.06); 39 | 25.60 (4.43); 65 | 25.16 (4.27); 184 | 23.79 (4.29); 22 | 25.66 (4.63); 134 | 25.31 (4.34); 23 | 25.23 (3.47); 49 | 25.43 (4.02); 139 | 25.05 (3.95); 17 | 25.37 (4.09); 98 |

*Note*. WOW/BFL= Women on Wheels/Bike for Life, GFL = Go for Life Games, GIW = Get Ireland Walking, MOM = Men on the Move, CON = Control; MVPA = moderate to vigorous physical activity, LiPA = light physical activity, Stand = time standing, Sed. Time = sedentary time during waking hours, PAGL = physical activity guidelines, BMI = body mass index, Waist Circ. = waist circumference, TUG = Timed Up and Go test, 6MWT = 6-Minute Walk Test, wellbeing = mental wellbeing.
